# Serum cytokines and neutrophil-to-lymphocyte ratio as predictive biomarkers of benefit from PD-1 inhibitors in gastric cancer

**DOI:** 10.3389/fimmu.2023.1274431

**Published:** 2023-10-31

**Authors:** Yidan Hou, Xiaoli Li, Yudan Yang, Hao Shi, Shaofang Wang, Ming Gao

**Affiliations:** Department of Oncology, The First Affiliated Hospital of Zhengzhou University, Zhengzhou, Henan, China

**Keywords:** gastric cancer, immune-checkpoint inhibitors, cytokines, predictive biomarker, blood cell ratio, overall survival, progression-free survival

## Abstract

**Background:**

Immunotherapy is significantly revolutionizing cancer treatment and demonstrating promising efficacy in gastric cancer (GC) patients. However, only a subset of patients could derive benefits from targeted monoclonal antibody therapy against programmed death receptor 1 (PD-1). This study aims to identify suitable serum cytokines and blood cell ratios as predictive biomarkers to aid in the selection of GC patients likely to benefit from PD-1 inhibitors.

**Materials and methods:**

This retrospective study included 41 GC patients who received PD-1 inhibitors combined with chemotherapy, 36 GC patients treated solely with chemotherapy, and 33 healthy controls. The study assessed the levels of seven cytokines: interleukin-2 (IL-2), IL-4, IL-6, IL-10, IL-17A, tumor necrosis factor-alpha (TNF-α), interferon-gamma (IFN-γ), and various inflammatory markers, including the neutrophil-to-lymphocyte ratio (NLR), total lymphocyte count (TLC), platelet-to-lymphocyte ratio (PLR), and lymphocyte-to-monocyte ratio (LMR). Measurements were obtained using the inpatient system. Univariate and multivariate Cox regression analyses were performed to evaluate the predictive significance of these hematologic parameters for clinical outcomes.

**Results:**

Levels of IL-6, IL-10, TNF-α, NLR, and PLR were significantly elevated in GC patients compared to healthy controls, while TLC and LMR were higher in the control group. Among the 41 patients receiving PD-1 inhibitors and chemotherapy, baseline IL-2 was associated with OS and PFS. Additionally, IL-6 and IL-17A correlated with OS, while NLR was linked to PFS (all P<0.05). These factors were identified as independent prognostic indicators in both univariate and multivariate analyses. Furthermore, almost all cytokine levels increased following the initiation of PD-1 inhibitor treatment.

**Conclusions:**

The introduction of PD-1 inhibitors alongside chemotherapy in GC impacts serum cytokine levels. IL-2, IL-6, IL-17A, and NLR exhibit potential as reliable circulating predictive biomarkers for identifying patients who may benefit from PD-1 inhibitors combined with chemotherapy.

## Introduction

Gastric cancer is a significant global health concern, ranking fifth in terms of incidence and fourth in mortality worldwide (1). Certain regions, such as Eastern Asia, Eastern Europe, and South America, have particularly high rates of gastric cancer cases. In mainland China, a considerable number of patients are diagnosed at an advanced stage due to low screening rates and subtle clinical symptoms, resulting in missed opportunities for surgery and poorer prognoses (2). Fortunately, the development of immunotherapy for gastric cancer has shown promising results, changing traditional treatment approaches.

The immune checkpoint is a vital element of the immune system, consisting of receptors found on the surface of immune cells that can either positively or negatively regulate immune responses. For example, PD-1, located on the surface of T cells, functions as a natural brake to control the excessive activity of cytotoxic T effector cells when it binds to its ligand PD-L1. PD-L1 is commonly found in both normal tissues and tumor cells, and their interactions help limit immune-mediated tissue damage and support tumor cells in evading the immune system (3). Immunotherapy using Immune Checkpoint Inhibitors (ICIs) has emerged as a promising approach in the treatment of various cancers. ICIs target the PD-1/PD-L1 pathway to boost the reactivity of anti-tumor T cells. Notably, several PD-1 inhibitors (Nivolumab, Pembrolizumab, Sintilimab, Camrelizumab, Tislelizumab) and PD-L1 inhibitors (Atezolizumab, Avelumab, Durvalumab) have received approval for cancer therapy and have demonstrated effectiveness in an expanding range of malignancies, including gastroesophageal, melanoma, and lung cancers (4). However, despite these significant advancements, a considerable proportion of gastric cancer patients receiving ICIs do not derive therapeutic benefits (5). Numerous clinical studies have been conducted to identify biomarkers that can predict which gastric cancer patients are likely to respond well to ICIs therapy. Some potential biomarkers include PD-L1 expression, tumor mutational burden (TMB), microsatellite instability/mismatch repair (MSI/MMR) status, Epstein-Barr virus (EBV) infection, circulating tumor DNA (ctDNA), and gut microbiota. However, their practical application in day-to-day clinical practice still requires further confirmation (6).

Recent research has been rapidly uncovering the mechanisms linking infection, innate immunity, inflammation, and cancer (7). Cytokines, produced by activated immune cells, play a crucial role in this linkage. Pro-inflammatory cytokines such as IL-1β, IL-8, IL-12, TNF-α, IFN-γ, and anti-inflammatory cytokines like IL-4 and IL-10 have dual functions, activating anti-tumorigenic actions of T cells while also participating in tumor malignant transformation, growth, invasion, and metastasis (8). Cytokines can activate anti-tumorigenic actions of T cells and also contribute to tumor growth, invasion, and metastasis (9). Moreover, systemic inflammatory response (SIR) indicators, such as NLR, PLR, LMR, and TLC, have been reported to be associated with the prognosis of certain cancers (10). Changes in cytokine expression levels and cell composition in the tumor microenvironment (TME) can potentially influence the efficacy of ICIs in various malignancies (11). Therefore, multiplex cytokine and blood cell analysis could yield valuable prognostic assessments in patients.

This study aims to examine the association between baseline and post-treatment peripheral cytokines and blood cells in GC patients who received PD-1 inhibitors combined with chemotherapy. The goal is to identify clinically significant predictive factors for the efficacy of immunotherapy in patients with gastric cancer.

## Materials and methods

### Patient characteristics

December 2022. Among them, 41 GC patients received PD-1 inhibitors in combination with chemotherapy (Cohort 1), while 36 GC patients underwent chemotherapy alone (Cohort 2). The 33 healthy controls exhibited good health without any indications of tumors, viral infections, diabetes, connective tissue diseases, or liver/kidney impairments. Inclusion criteria for the 77 patients included: 1) histopathological confirmation of gastric cancer at stage II-IV according to the American Joint Committee on Cancer (AJCC); 2) receiving PD-1 inhibitors combined with chemotherapy (Cohort 1) or chemotherapy alone (Cohort 2) for a minimum of 3 cycles; 3) regular tumor assessments every 2 treatment courses using imaging evaluations, with Overall Survival (OS) and Progression-Free Survival (PFS) times recorded based on imaging results and follow-up phone calls; 4) blood samples collected for cytokine and blood cell analysis when tumor progression or response was observed; 5) physical condition scored according to the Eastern Cooperative Oncology Group guidelines (ECOG) ranging from 0 to 3 (12), and no dysfunction in vital organs detected. This study adhered to the principles outlined in the World Medical Association’s Declaration of Helsinki and received approval from the Medical Ethical Committee of our hospital. Since only anonymous data were used for this retrospective study, the ethics committee waived the requirement for informed consent.

### Treatment

In Cohort 2, 36 GC patients were treated: 19 received first-line Sox (Oxaliplatin, Teggio) chemotherapy, 15 received first-line Sox combined with albumin-bound paclitaxel, and 2 received first-line Xelox (oxaliplatin and capecitabine). The median number of cycles for the first-line chemotherapy was 5, with a range from 3 to 10, and no subsequent PD-1 inhibitor treatment was administered. In Cohort 1, 41 patients were included: 14 received PD-1 inhibitors as part of the first-line therapy, while 27 received PD-1 inhibitors during subsequent-line therapy. The PD-1 inhibitors used were Sintilimab, Camrelizumab, and Tislelizumab, combined with chemotherapy over a 21-day cycle. The chemotherapy regimen was consistent with the description above. The median number of chemoimmunotherapy cycles was 5, with a range from 3 to 14.

### Analysis of survival

Tumor assessments were performed after every two treatment courses using various imaging techniques such as CT, ultrasound, MRI, or PET-CT. The evaluation was conducted following the Response Evaluation Criteria of Solid Tumors 1.1 (RECIST1.1) criteria (13). PFS was calculated from the initiation of anti-tumor therapy to the date of disease progression. On the other hand, OS was measured from the date of the first treatment dose until death from any cause.

### Blood sample collection and measurements

Plasma samples were collected from the patients before the first treatment and at the time of disease remission or progression. These samples were then centrifuged at 1000 g for 10 min at 4°C. After centrifugation, the supernatant (serum) was immediately extracted and analyzed on the spot or divided into aliquots and stored frozen at −80°C. Cytokine levels were assessed using the Human Cytokine 12 Plex Kit (Beijing ACRO Biosystems, catalog number: CRS- A002/A017/B001/B003/B005/B008) at the clinical laboratory department of our hospital. The panel of measured cytokines included IL-2, IL-4, IL-6, IL-10, IFN-γ, TNF-α, and IL-17A. Beyond that, blood routine examination was achieved by flow cytometry, NLR, PLR, and LMR were then calculated as the total neutrophil counts divided by the lymphocyte counts, platelet counts divided by the TLCs, and the TLCs divided by the total monocyte counts, respectively.

### Cytokine cut-off value calculation

To assess the correlation between baseline blood parameters and survival, we categorized the baseline blood parameters into high-level and low-level groups. This categorization was based on either the median value or the optimal cut-off value (**Tables S1-2**). To ascertain the most suitable cut-off value for the studied indicators, we employed the web-based software X-tile (**Table S3-4**).

### Statistical analyses

Patient characteristics underwent analysis using descriptive statistical methods. Continuous variables were summarized using medians and quartiles, and comparisons were conducted using the Mann-Whitney U test and the Kruskal-Wallis test. Categorical variables were presented as numbers (%) and analyzed using the chi-squared test and Fisher’s exact test. For the evaluation of independent prognostic factors, both univariate and multivariate analyses were performed. Hazard ratios (HRs) and 95% confidence intervals (CIs) were reported. In the multivariable model, only elements with a p-value of <0.1 from the univariate analysis were incorporated. The significance threshold for multivariate analyses was set at P < 0.05. OS and PFS were illustrated using the Kaplan-Meier method, and the log-rank test was employed to compare the survival curves. All statistical analyses were executed using SPSS version 26.0 software, and the figures were generated using GraphPad Prism version 8.0.

## Results

### Patients’ characteristics and survival outcomes

**Table 1** presents the clinical characteristics and pre-treatment blood parameters of the 77 patients diagnosed with GC. Cohort 1 consisted of a higher percentage of patients in stages III-IV of the TNM classification (82.93% vs. 58.33%) and more patients who had not undergone gastric surgery (56.10% vs. 22.22%) compared to Cohort 2. The level of IL-6 was found to be higher in Cohort 1 than in Cohort 2 (12.06 vs. 4.85). Patients in Cohort 1, who received chemoimmunotherapy, experienced a significantly better PFS of 10.67 months compared to 8.1 months in Cohort 2 (p = 0.003). Additionally, Cohort 1 also showed an improved OS of 15.7 months compared to 10.83 months in Cohort 2 (p = 0.021). No statistically significant differences were observed between Cohort 1 and Cohort 2 regarding age, sex, ECOG score, presence of other chronic diseases (diabetes, hypertension, cardiopathy), history of smoking, and family history of cancer (p > 0.05).

**Table 1:** Characteristics of patients at baseline.

Clinical characteristics		GC patients (n=77) n (%)	Cohort 1 (n=41) n (%)	Cohort 2 (n=36) n (%)	P
**Gender**	male	53 (68.831%)	26 (63.415%)	27 (75.000%)	0.273
	female	24 (31.169%)	15 (36.585%)	9 (25.000%)	
**Age**	<60	41 (53.247%)	24 (58.537%)	17 (47.222%)	0.321
	≥60	36 (46.753%)	17 (41.463%)	19 (52.778%)	
**ECOG score**	≤2	63 (81.818%)	34 (82.927%)	29 (80.556%)	0.788
	>2	14 (18.182%)	7 (17.073%)	7 (19.444%)	
**TNM stage**	II	22 (28.571%)	7 (17.073%)	15 (41.667%)	**0.017**
	III-IV	55 (71.429%)	34 (82.927%)	21 (58.333%)	
**Surgery history**	Yes	46 (59.740%)	18 (43.902%)	28 (77.778%)	**0.002**
	No	31 (40.260%)	23 (56.098%)	8 (22.222%)	
**Smoked**	Yes	27 (35.065%)	15 (36.585%)	12 (33.333%)	0.765
	No	50 (64.935%)	26 (63.415%)	24 (66.667%)	
**family cancer history**	Yes	14 (18.182%)	6 (14.634%)	8 (22.222%)	0.389
	No	63 (81.818%)	35 (85.366%)	28 (77.778%)	
**other chronic disease**	Yes	20 (25.974%)	10 (24.390%)	10 (27.778%)	0.735
	No	57 (74.026%)	31 (75.610%)	26 (72.222%)	
**mPFS** (month)	median	8.87	10.67	8.1	**0.003**
**mOS** (month)	median	14.83	15.7	10.83	**0.021**
**IL-2**	median[Q1, Q3]	1.740[1.210,2.590]	1.960[1.420,2.600]	1.490[1.110,2.260]	0.213
**IL-4**	median[Q1, Q3]	1.990[0.910,3.310]	2.370[1.020,3.310]	1.930[0.800,3.080]	0.444
**IL-6**	median[Q1, Q3]	6.100[3.970,14.190]	12.060[5.020,18.060]	4.850[2.980,8.280]	**<0.001**
**IL-10**	median[Q1, Q3]	2.690[1.870,3.790]	2.860[1.870,3.840]	2.650[1.890,3.650]	0.748
**TNF-α**	median[Q1, Q3]	1.940[1.300,2.700]	1.870[1.470,2.550]	2.230[1.230,2.910]	0.537
**IFN-γ**	median[Q1, Q3]	2.060[1.460,2.590]	2.110[1.500,2.560]	1.940[1.460,2.620]	0.736
**IL-17A**	median[Q1, Q3]	5.680[2.900,9.260]	5.680[2.340,9.890]	5.720[3.190,8.910]	0.779
**TLC**	median[Q1, Q3]	1.320[1.040,1.700]	1.310[1.000,1.790]	1.370[1.200,1.690]	0.721
**NLR**	median[Q1, Q3]	2.338[1.571,3.444]	2.600[1.692,4.500]	2.338[1.571,2.628]	0.234
**PLR**	median[Q1, Q3]	149.231[109.924,209.375]	149.231[109.924,205.833]	149.693[114.557,223.171]	0.732
**LMR**	median[Q1, Q3]	3.478[2.370,4.238]	3.462[2.167,4.238]	3.714[2.726,3.953]	0.713

Eastern Cooperative Oncology Group Performance Status (ECOG PS). P < 0.05 was considered statistically significant and shown in bold type.

### Comparison of baseline blood parameters between GC patients and healthy individuals

**Figure 1:**
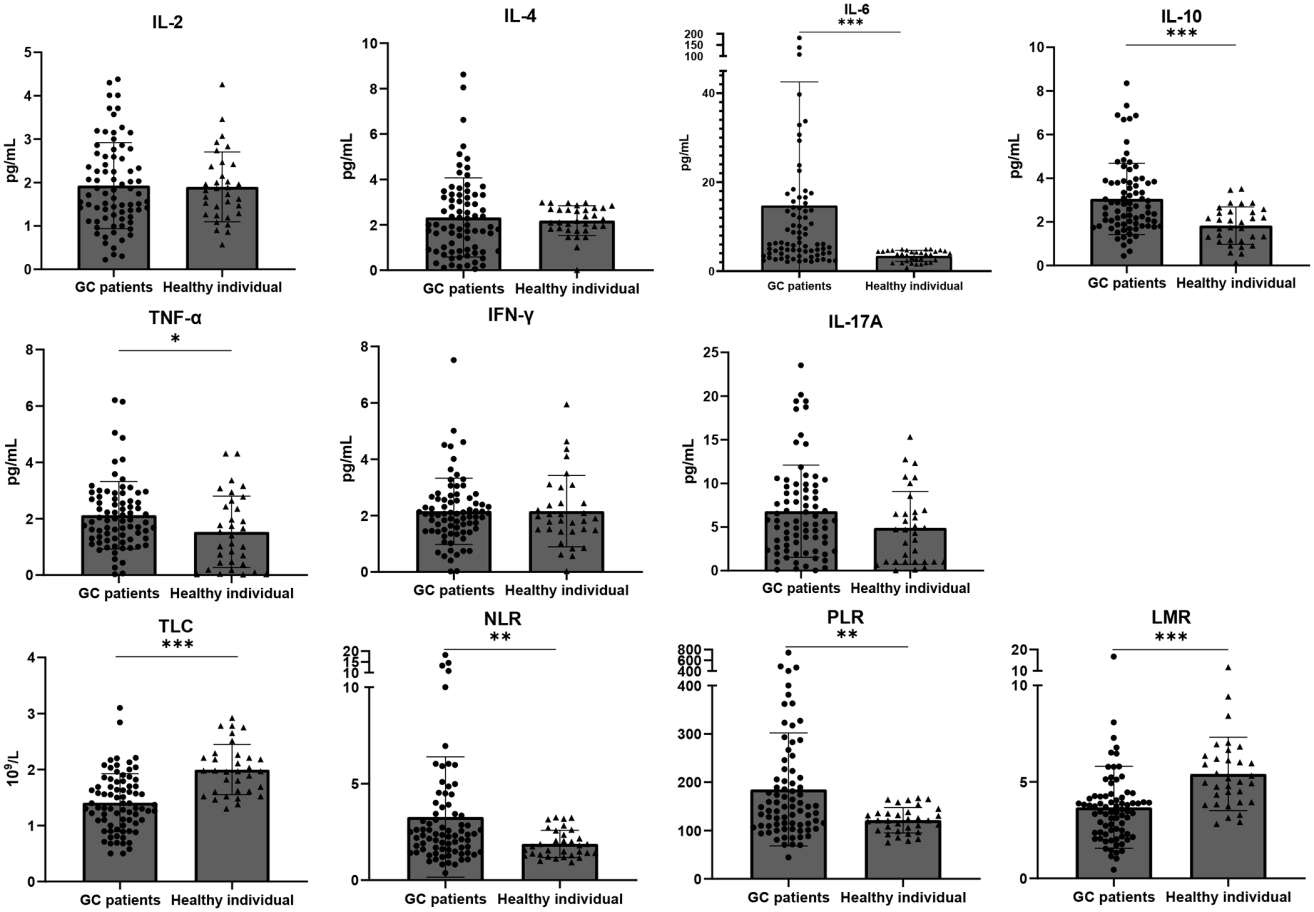
Baseline IL-6, IL-10, TNF-α, NLR and PLR are higher, TLC and LMR are lower in GC patients than in healthy individuals. Dot plots show the difference of baseline blood parameters between healthy volunteers (n = 33) and GC patients (n = 77). The top of the grey box shows the median value. All cytokines except for IL-2 and IL-4 were higher in GC patients, but only in the case of IL-6 (p<0.0001), IL-10 (p<0.0001) and TNFα (p = 0.021) these differences were statistically significant. TLC (p<0.0001) and LMR (p<0.0001) were higher in healthy individuals while NLR(p=0.0085 )and PLR (p=0.0034)were higher in GC patients. Error bars show the interquartile range. *p < 0.05, **p < 0.01, and ***p < 0.001.

To clarify the significance of cytokines in GC diagnosis, we included 33 healthy participants. As displayed in **Table S5**, there was no marked difference in age and gender distribution between the healthy controls and GC patients (p > 0.05), making subsequent results comparable. **Figure 1** reveals that, aside from IL-2 and IL-4, levels of all other cytokines were elevated in GC patients compared to healthy individuals. Specifically, the differences in IL-6, IL-10, and TNF-αwere statistically significant (p<0.0001, p<0.0001, p = 0.021, respectively). It is worth highlighting that every blood cell component ratio studied exhibited statistical differences between the two cohorts. In healthy individuals, both TLC and LMR were higher (p<0.0001, p<0.0001), while GC patients had elevated NLR and PLR (p = 0.0085, p = 0.0034) (**Table S6**).

### Correlation between blood indexes and clinical features in GC patients

As illustrated in **Table S7**, females exhibited notably higher baseline levels of IL-6 and PLR, while their NLR was significantly lower (p = 0.032, p = 0.046, p = 0.003, respectively). Elevated IL-6 levels were also observed in patients without a family history of cancer (p = 0.012) and in those aged above 60 years (p = 0.048). Patients who underwent gastric surgery had significantly increased levels of IFN-γ and NLR (p = 0.009, p = 0.017). Patients with an ECOG score of ≤2 had a notably raised TLC (p = 0.048). Conversely, LMR was distinctly lower in patients diagnosed with primary diseases such as hypertension, diabetes, and stroke (p = 0.028). There were no statistically significant differences in cytokine levels based on clinical stages or smoking histories (p > 0.05).

### Associations between blood indexes and survival outcomes

**Figure 2:**
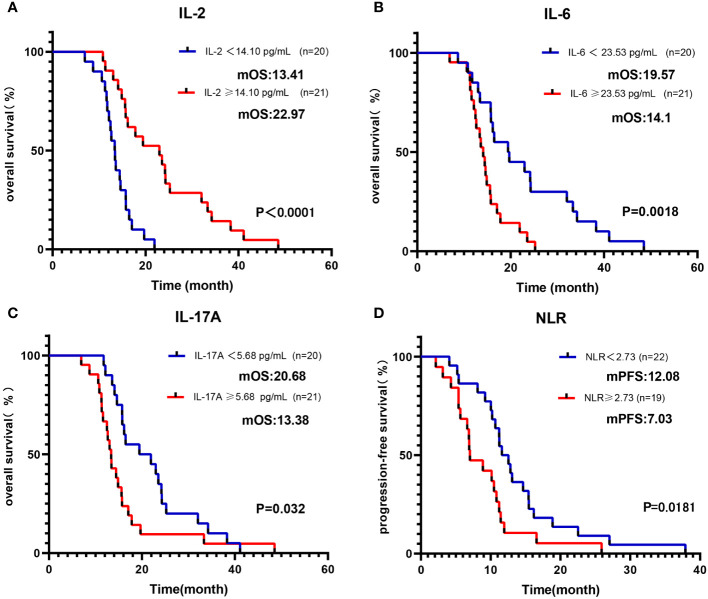
Kaplan-Meier curve of OS/PFS of Cohort 1 patients, grouped by median of baseline blood parameters. Kaplan-Meier OS curves according to baseline median of **(A)** IL-2; **(B)** IL-6; **(C)** IL-17A. Kaplan-Meier PFS curves according to baseline median of **(D)** NLR.

Initially, we categorized baseline blood parameters into a high-level group and a low-level group based on the median value. As outlined in **Table 2**, univariate analysis revealed a significant association between OS and several factors, including IL-2, IL-6, IFN-γ, IL-17A, NLR, and ECOG (all p < 0.05). To account for other potential influences on survival outcomes, a multivariable Cox regression analysis was conducted. It confirmed that the IL-2-high group had an improved OS, whereas the IL-6-high and IL-17A-high groups exhibited reduced OS (all p < 0.05) (**Figures 2A–C**). Regarding PFS, the univariate analysis indicated significant associations with IL-2, IL-4, IL-6, IL-10, IFN-γ, and NLR (all p < 0.1). Subsequent multivariate analysis confirmed that the NLR-high group had a reduced PFS (p <0.01) (**Figure 2D** **).** In a similar manner, we conducted an analysis of the prognostic impact of blood parameters (categorized by the median) after the initial 2 treatment cycles. The multivariable regression analysis revealed that the IL-6-low group exhibited an enhanced OS and PFS, whereas the IL-2-high groups showed increased OS. Conversely, the IL-17A-high group demonstrated a diminished PFS (all with p < 0.05) (**Table S8**).

**Table 2:** Univariate and Multivariate analysis for PFS and OS of Cohort 1 baseline blood parameters grouped by median.

	OS				PFS			
Characteristics	univariate analysis		multivariate analysis		univariate analysis		multivariate analysis	
	HR (95% CI)	P	HR (95% CI)	P	HR (95% CI)	P	HR (95% CI)	P
**IL-2**	**0.217 (0.098-0.478)**	**0.000**	**0.382 (0.165-0.888)**	**0.025**	**0.432 (0.223-0.838)**	**0.013**	0.702 (0.294-1.674)	0.425
**IL-4**	0.745 (0.396-1.401)	0.361	**-**	**-**	**0.547 (0.287-1.041)**	**0.066**	0.73 (0.333-1.599)	0.431
**IL-6**	**2.944 (1.453-5.965)**	**0.003**	**3.018 (1.367-6.666)**	**0.006**	**2.212 (1.145-4.273)**	**0.018**	1.882 (0.866-4.089)	0.110
**IL-10**	0.74 (0.393-1.396)	0.353	**-**	**-**	**0.516 (0.265-1.002)**	**0.051**	0.668 (0.311-1.437)	0.302
**TNF-α**	0.88 (0.46-1.683)	0.699	**-**	**-**	0.919 (0.49-1.722)	0.791	**-**	**-**
**IFN-γ**	**0.355 (0.174-0.725)**	**0.004**	0.553 (0.251-1.218)	0.142	**0.567 (0.296-1.084)**	**0.086**	0.79 (0.372-1.677)	0.540
**IL-17A**	**1.978 (1.049-3.729)**	**0.035**	**2.143 (1.077-4.265)**	**0.030**	1.382 (0.739-2.587)	0.311	**-**	**-**
**TLC**	0.956 (0.501-1.824)	0.891	**-**	**-**	1.454 (0.727-2.908)	0.289	**-**	**-**
**NLR**	**2.162 (1.137-4.111)**	**0.019**	2.022 (0.981-4.166)	0.056	**2.38 (1.241-4.563)**	**0.009**	**2.886 (1.418-5.876)**	**0.003**
**PLR**	1.052 (0.559-1.981)	0.876	**-**	**-**	0.946 (0.508-1.763)	0.862	**-**	**-**
**LMR**	1.156 (0.607-2.203)	0.660	**-**	**-**	0.874 (0.467-1.635)	0.673	**-**	**-**
**gender**	0.921 (0.479-1.768)	0.804	**-**	**-**	0.875 (0.459-1.667)	0.685	**-**	**-**
**age**	0.74 (0.39-1.403)	0.357	**-**	**-**	0.968 (0.504-1.86)	0.922	**-**	**-**
**ECOG (>2)**	**10.172 (3.453-29.966)**	**0.000**	**7.481 (2.19-25.548)**	**0.001**	1.659 (0.723-3.81)	0.233	**-**	**-**
**TNM stage (III-IV)**	1.267 (0.526-3.055)	0.598	**-**	**-**	0.953 (0.391-2.322)	0.915	**-**	**-**
**surgery history**	1.33 (0.699-2.529)	0.385	**-**	**-**	0.957 (0.512-1.79)	0.891	**-**	**-**
**other chronic basic diseases**	1.001 (0.484-2.069)	0.999	**-**	**-**	1.072 (0.521-2.208)	0.850	**-**	**-**
**smoked**	0.903 (0.47-1.734)	0.758	**-**	**-**	0.97 (0.508-1.852)	0.927	**-**	**-**
**family cancer history**	1.373 (0.563-3.349)	0.486	**-**	**-**	0.765 (0.319-1.833)	0.548	**-**	**-**

Baseline blood parameters were grouped by the median. HR, hazard ratios; CI, confidence interval. basic disease (diabetes, hypertension, cardiopathy). Elements with a p-value of <0.1 in the univariate analysis and with a p-value of <0.05 in the multivariate analysis were in bold type.

**Figure 3:**
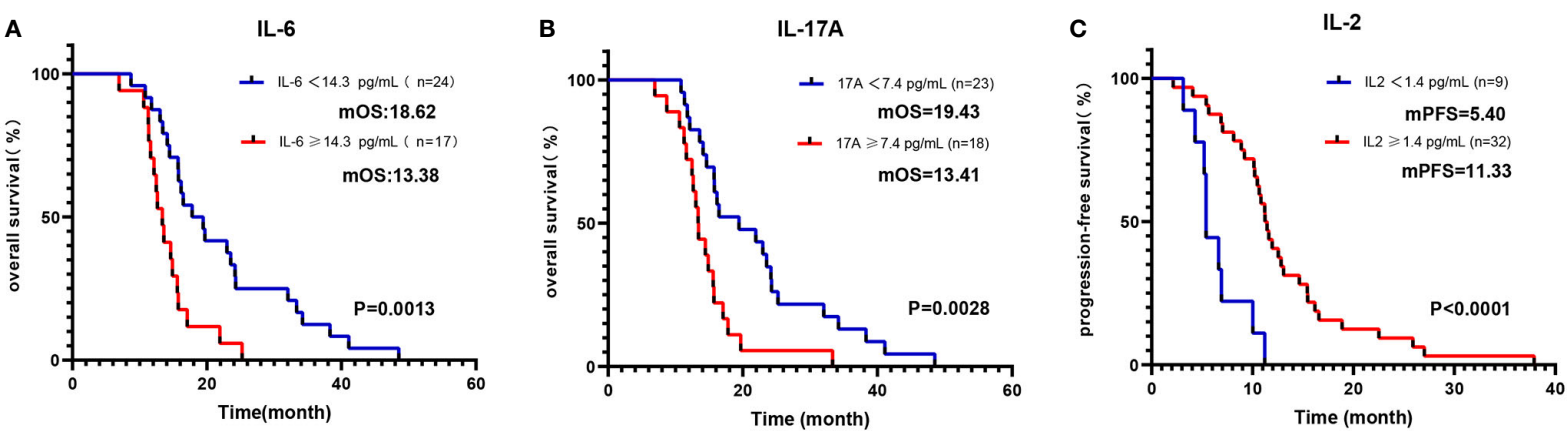
Kaplan-Meier curve of OS/PFS of Cohort 1 patients, grouped by cut-off values of baseline blood parameters. Kaplan-Meier OS curves according to baseline cut-off values of **(A)** IL-6; **(B)** IL-17A. Kaplan-Meier PFS curves according to baseline cut-off values of **(C)** IL-2.

As detailed in **Table 3**, the blood parameters of Cohort 1 were divided into high-level and low-level groups based on a cut-off value. Univariate analysis identified significant links between OS and parameters such as IL-2, IL-6, TNF-α, IFN-γ, IL-17A, NLR, and ECOG (all p <0.05). Further multivariate analysis verified that the IL-6-high and IL-17A-high groups had diminished OS (all p < 0.05) (**Figures 3A, B**). Similarly, univariate analysis revealed a significant relationship between PFS and variables like IL-2, IL-4, IL-6, IL-10, TNF-α, IFN-γ, NLR, and LMR (all p < 0.1). Upon multivariate assessment, the IL-2-high group was found to have a superior PFS (all p < 0.05) (**Figure 3C**). In a parallel manner, the Cox regression analysis of blood parameters (categorized by cut-off value) after the initial 2 treatment cycles indicated that the IL-2-high and IL-6-low groups experienced enhanced overall survival (OS) and progression-free survival (PFS), while the IL-17A-high groups displayed decreased PFS (all p < 0.01) (**Table S9**).

**Table 3:** Univariate and Multivariate analysis for PFS and OS of Cohort 1 baseline blood parameters grouped by cut-off value.

	OS				PFS			
Characteristics	univariate analysis		multivariate analysis		univariate analysis		multivariate analysis	
	HR (95% CI)	P	HR (95% CI)	P	HR (95% CI)	P	HR (95% CI)	P
**IL-2**	**0.215 (0.097-0.474)**	**0.000**	0.735 (0.267-2.026)	0.552	**0.195 (0.083-0.456)**	**0.000**	**0.354 (0.127-0.983)**	**0.046**
**IL-4**	0.713 (0.378-1.346)	0.297	–	–	**0.47 (0.243-0.909)**	**0.025**	0.62 (0.287-1.34)	0.224
**IL-6**	**2.969 (1.489-5.92)**	**0.002**	**3.092 (1.204-7.943)**	**0.019**	**1.815 (0.923-3.569)**	**0.084**	2.114 (0.935-4.78)	0.072
**IL-10**	0.65 (0.337-1.254)	0.199	–	–	**0.423 (0.209-0.859)**	**0.017**	0.511 (0.226-1.156)	0.107
**TNF-α**	**0.278 (0.111-0.701)**	**0.007**	0.697 (0.189-2.573)	0.588	**0.45 (0.183-1.107)**	**0.082**	1.828 (0.53-6.301)	0.339
**IFN-γ**	**0.355 (0.174-0.725)**	**0.004**	0.475 (0.179-1.262)	0.135	**0.45 (0.213-0.949)**	**0.036**	0.414 (0.17-1.006)	0.051
**IL-17A**	**2.704 (1.378-5.306)**	**0.004**	**2.715 (1.156-6.375)**	**0.022**	1.382 (0.739-2.587)	0.311	–	–
**TLC**	0.703 (0.332-1.491)	0.359	–	–	1.218 (0.63-2.354)	0.558	–	–
**NLR**	**2.967 (1.301-6.766)**	**0.010**	2.036 (0.758-5.463)	0.158	**2.22 (1.149-4.29)**	**0.018**	1.645 (0.653-4.141)	0.291
**PLR**	0.775 (0.374-1.606)	0.493	–	–	0.716 (0.352-1.46)	0.359	–	–
**LMR**	0.508 (0.213-1.211)	0.127	–	–	**0.54 (0.274-1.065)**	**0.075**	0.575 (0.207-1.594)	0.287
**gender**	0.921 (0.479-1.768)	0.804	–	–	0.875 (0.459-1.667)	0.685	–	–
**age**	0.74 (0.39-1.403)	0.357	–	–	0.968 (0.504-1.86)	0.922	–	–
**ECOG (>2)**	**10.172 (3.453-29.966)**	**0.000**	**7.546 (2.281-24.966)**	**0.001**	1.659 (0.723-3.81)	0.233	–	–
**TNM stage** **(III-IV)**	1.267 (0.526-3.055)	0.598	–	–	0.953 (0.391-2.322)	0.915	–	–
**surgery history**	1.33 (0.699-2.529)	0.385	–	–	0.957 (0.512-1.79)	0.891	–	–
**other chronic basic disease**	1.001 (0.484-2.069)	0.999	–	–	1.072 (0.521-2.208)	0.850	–	–
**smoked**	0.903 (0.47-1.734)	0.758	–	–	0.97 (0.508-1.852)	0.927	–	–
**family cancer history**	1.373 (0.563-3.349)	0.486	–	–	0.765 (0.319-1.833)	0.548	–	–

Baseline blood parameters were grouped by cut-off value. Elements with a p-value of <0.1 in the univariate analysis and with a p-value of <0.05 in the multivariate analysis were in bold type.

Interestingly, these relationships between blood parameters and clinical outcomes were exclusive to Cohort 1. When focusing on Cohort 2, where patients underwent only chemotherapy, these associations were not evident (**Tables S10-11**). This implies that baseline serum IL-2, IL-6, IL-17A, and NLR can independently forecast the efficacy of PD-1 inhibitors in GC patients.

### Dynamic changes of cytokines once treatment was initiated in each cohort

**Figure 4:**
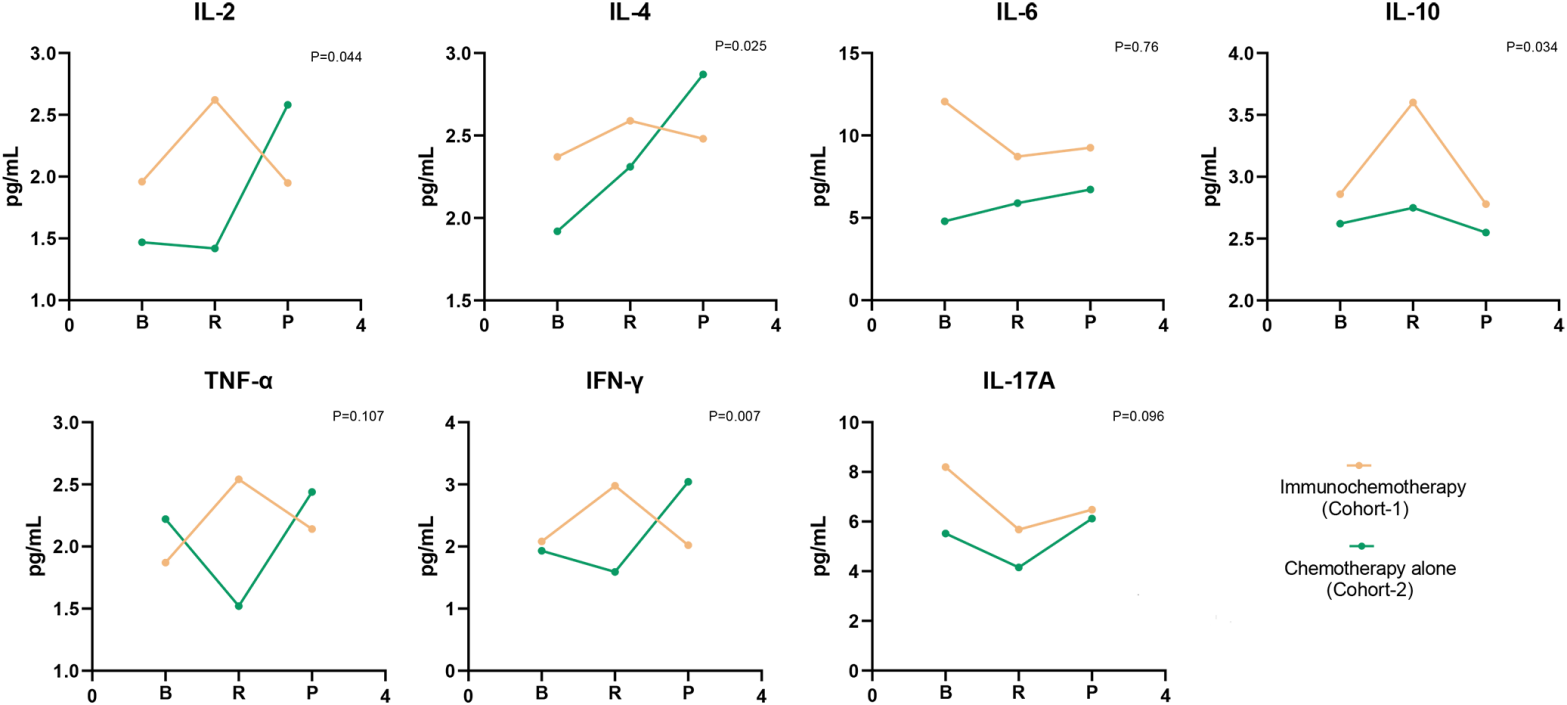
Evolution of cytokine levels in patients of two Cohorts. Values corresponded to the median of cytokine titers, and p values were obtained taking into account the difference of cytokine levels in the baseline and response period. B, baseline; R, response, include first complete response (CR) and partial response (PR); P, progression.

**Figure 5:**
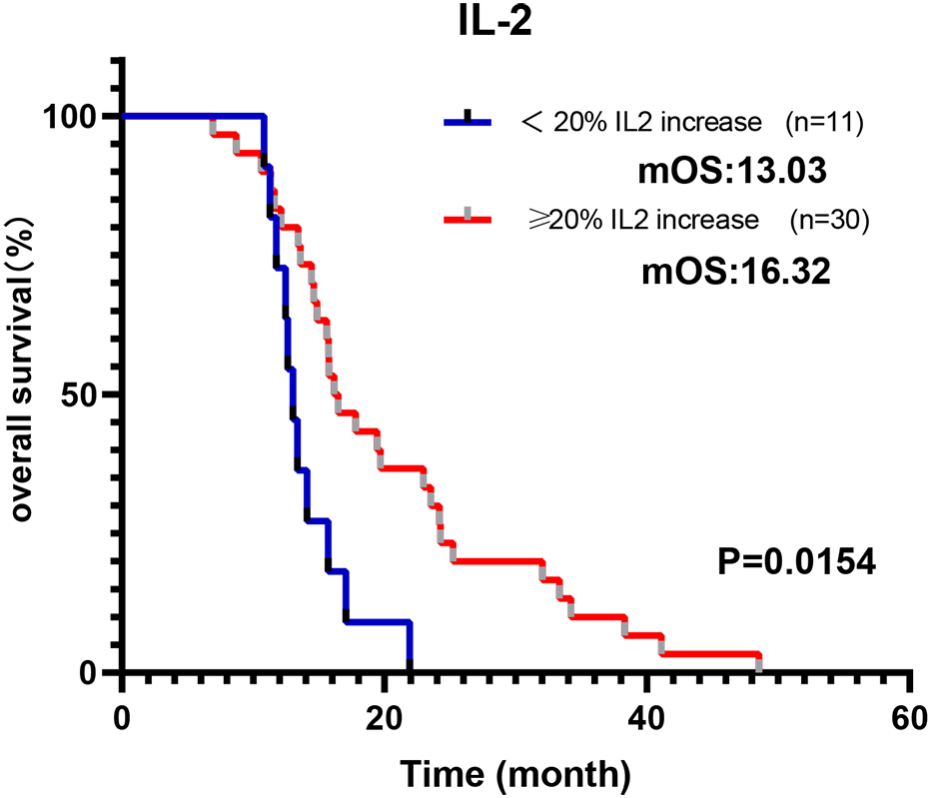
Modulation of IL-2 during immunochemotherapy treatment predicts a better prognosis. Cohort 1 patients whose IL-2 increased more than 20% from baseline to response indicate a longer OS.

As depicted in **Figure 4**, there was a general elevation from baseline to the moment the tumor exhibited its first complete response (CR) or partial response (PR) in all cytokines, with the exceptions being IL-6 and IL-17A in Cohort 1. Notably, the levels of IL-2, IL-4, IL-10, and IFN-γ were statistically significantly increases (p = 0.044, p = 0.025, p = 0.034, p = 0.007, respectively). In contrast, Cohort 2 displayed a decline in IL-2, TNF-α, IFN-γ, and IL-17A from baseline to response. While IL-4, IL-6, and IL-10 demonstrated a rise from baseline to tumor response, none of these changes reached statistical significance. We calculated the percentage variations in cytokine levels from baseline to response to determine if these quantitative shifts during treatment correlated with survival outcomes. As illustrated in **Figure 5**, individuals in Cohort 1, where IL-2 levels increased by over 20% from baseline to response, showed a considerably improved OS (16.32 m vs. 13.03 m; p = 0.0154). This trend in IL-2 variation was also observed in Cohort 2 patients, but it did not maintain statistical significance (13.58 m vs. 12.49 m, p = 0.6537). We additionally computed the percentage variations in cytokine levels from baseline to the timepoint following 2 treatment cycles, but we did not observe any consistent trend.

## Discussion

The detection of cytokines and blood cell parameters is preferable to other biomarkers due to their widespread use and minimally invasive sampling technique. In this study, we had access to two groups of patients treated either with immunochemotherapy or solely with chemotherapy. This allowed us to assess the biological impacts of incorporating Immune ICIs. Only associations that were notably significant in Cohort 1, distinct from those observed in Cohort 2 (chemotherapy-only group), were considered indicative of the effects related to ICIs. We employed statistical analysis to determine whether baseline levels and variations in cytokines and blood cell parameters could predict the efficacy of immunotherapy across different treatment outcomes. Initially, we compared baseline cytokine levels of GC patients against those found in healthy subjects. One significant observation was the universally elevated cytokine levels in GC patients, with the exceptions being IL-2 and IL-4. Notably, levels of IL-6, IL-10, and TNF-α were markedly increased in the GC patient group. As depicted in **Figure 1**, there were no significant differences in the levels of IL-2, IL-4, INF-γ, and IL-17A between GC patients and healthy controls. IL-2 is primarily secreted by T cells (14), and our study observed a decrease in the total lymphocyte count among gastric cancer patients. Consistent with our findings, Mohammad et al. (15), reported no significant differences in IL-2 levels between gastric cancer patients and healthy controls. Furthermore, another study noted that patients with gastric cancer stage III or IV exhibited elevated levels of IL-2, while there was no distinction in the serum levels of IL-2 between patients with gastric cancer stage I or II and healthy controls (16), which aligns with our findings in **Table S7**. Increased IL-4 levels have been frequently observed in various types of cancers. However, the evidence regarding the pro- or antitumoral role of IL-4 is conflicting, and this function is closely linked to IL-4 levels and its interaction with other immunological modulators (17). IFN-γ, produced by numerous immune cell subsets (including T cells, natural killer cells, B cells, and others), possesses both pro-tumor and anti-tumor activities (18). Nitu et al. reported that no significant differences existed in the concentration of IFN-γ between patients and healthy controls (19), which is consistent with our findings. Norma et al. also identified that circulating levels of IL-6 and IL-10 were discernibly higher in GC patients compared to a healthy control group (20), aligning with our results. Numerous studies indicate the pivotal role of IL-6 in a variety of malignancies (21–23). Elevated serum IL-6 concentrations have been documented in several solid tumors, including those of the lung, breast, pancreas, and stomach (24). The STAT3 pathway, when activated by IL-6, up-regulates the expression of cyclins and down-regulates the expression of the cyclin-dependent kinase (Cdk) inhibitor p21. This mechanism consequently promotes tumor cell cycle progression, leading to metastasis and tumor cell proliferation (25). Additionally, IL-6 has been reported to prevent cellular senescence by increasing telomerase activity, thereby promoting tumor growth (26). Studies indicate that IL-10 primarily inhibits the differentiation and antigen-presenting properties of DCs (dendritic cells) during the early stages of immune response (27). As a result, IL-10 significantly suppresses the production of IL-2 from antigen-presenting cells. In the absence of Th1-associated cytokines (like IL-2), the T-cell-mediated response is inevitable (28). While TNF-α, a pro-inflammatory cytokine, has been linked to promoting tumor metastasis and correlated with advanced cancer stages (29–31), its presence in cancers has also been associated with immune suppression. Animal model research further supports TNF-α’s role in promoting tumor growth and malignancy (32–35). Conversely, there are reports suggesting the benefits of the potent pro-inflammatory cytokine (TNF-α) in cancer treatments, especially given its recognition as a major factor in the anti-tumor activities of Coley’s toxins (36). In this study, both NLR and PLR were statistically elevated in GC patients compared to healthy controls. The neutrophil-to-lymphocyte ratio in peripheral blood reflects the balance between systemic inflammation and immunity. Consistent with our results, Mishra et al. discovered that the NLR is higher in cancer patients and its elevated level is linked to a worse.

In SIR studies, elevated NLR levels after ICI treatment have been linked to reduced survival rates in advanced esophagus cancer and lung cancer (10, 37). Consistent with these findings, our patients with a pre-treatment NLR above the median demonstrated a notably worse PFS. This negative correlation may be indicative of the interplay between intense inflammation and compromised immune function (38). While some studies suggest that a higher PLR corresponds to a worse prognosis in lung cancer patients (38), there is a dearth of research examining whether PLR, TLC, and LMR values differ between cancer patients and healthy individuals.

Cytokines represent a broad category of intercellular signaling proteins that play a pivotal role in almost every aspect of human immunology. However, the interaction of cytokine signaling activities is highly complex due to the redundancy and pleiotropy exhibited by cytokines. Moreover, there exists an intricate network of “cytokine cascades,” wherein the expression of a specific cytokine gene is invariably influenced by other cytokines (39). Cytokines are subject to regulation through various mechanisms. For instance, the anti-inflammatory cytokine IL-10 can suppress the expression of TNF-α and IFN-γ, a process referred to as feedback inhibition (40). IL-4, on the other hand, can suppress the production of IFN-γ by T cells, a phenomenon known as antagonism (41), IL-2, conversely, can enhance the production of IFN-γ (42), and IL-17A can synergistically stimulate TNF-α-induced IL-8 production (43).

To explore the prognostic and predictive role of cytokines, we examined the baseline and variations in cytokine levels and assessed their influence on patient outcomes across both cohorts. Cohort 2 had a higher number of patients in the early stages, and more had undergone radical surgery, which is traditionally considered a positive indicator for survival. However, the better OS in Cohort 1 implies that immunotherapy plays a more pivotal role in enhancing survival. A comparative analysis of the two cohorts allowed us to discern the specific effects associated with ICIs. Based on our findings, IL-2 can be perceived as a predictor of favorable response to ICIs. Higher baseline levels of IL-2 correlated with a significantly extended PFS and OS in Cohort 1, a distinction not observed in Cohort 2. IL-2 is a cytokine important in T-cell proliferation and promoting immune responses, as well as in increasing the activity of natural killer cells (44). Garrelds et al. identified that mice deficient in IL-2 are more prone to gastrointestinal inflammation, resembling human ulcerative colitis (45). Ren et al. documented that combining IL-2 with anti-PD-1 helps overcome tumor resistance to ICIs in mice by reactivating intratumoral CD8+ T cells rather than CD4+ Treg cells (46). Similarly, Ewan A et al. reported a two-year remission resulting from combined anti-PD-1 and intralesional IL-2 therapy in two patients with locoregional metastatic melanoma. This impressive response was partly due to an altered tumor microenvironment, including increased PD-L1 expression and CD8 T cell infiltration (47). Moreover, as shown in **Figure 5**, patients of Cohort-1 whose IL-2 increased more than 20% from baseline as a response, had a longer OS, which conforms to our preceding view.

IL-6 seems to be a predictor of resistance to ICIs, as patients with higher levels of this factor were found to have significantly worse OS. These observations perfectly agree with the study by Yu et al., who reported that increased circulating levels of IL-6 are associated with poor outcomes in liver cancer patients who received therapy with PD-1 inhibitors (48). IL-6 is a pro-inflammatory cytokine that may contribute to tumor progression by stimulating angiogenesis, invasion, and metastasis (8, 49). In some studies, increased IL-6 serum levels were reported to be associated with metastasis and poor prognosis in prostate, ovarian, and gastrointestinal cancers (21, 50, 51). Tsukamoto et al. indicated that increased IL-6 levels could indicate decreased efficacy of PD-1 blockade in patients with melanoma, and IL-6 blockade augments PD-L1 expression on tumor cells (52). Consistently, a study using IL-6-deficient mice bearing a murine colon cancer cell line found that the lack of IL-6 enhances the induction of effector T cells and inhibits tumorigenesis. Additionally, PD-L1 expression levels on tumor cells were significantly increased in the IL-6-deficient mice compared with wild-type mice (53). These findings strongly indicate the negative immune role of IL-6, especially in patients receiving ICIs.

IL-17A is a prominent member of the IL-17 family of pro-inflammatory cytokines. Prior research has reported its upregulation in the serum and tumors of GC patients. Kang et al. suggested that IL-17A promotes gastric carcinogenesis by regulating the IL-17RC/NF-κB/NOX1 pathway (54). However, it is worth noting that Karl et al. (55) found decreased IL-17A levels in esophageal adenocarcinoma patients when compared to healthy controls. In our study, we observed a less pronounced elevation of IL-17A in GC patients in comparison to healthy controls (as shown in **Figure 1**). Furthermore, our study revealed that GC patients with lower levels of IL-17A experienced improved OS, as demonstrated in **Figures 2C**, **3B**. Interestingly, IL-17A exhibited a noticeable decline from baseline to the point of maximum tumor remission. Accumulating evidence indicates that IL-17A activity may contribute to resistance to anti-tumor immunity and play a role in therapeutic failure. It is reported that the IL-17A signaling pathway can enhance the immunosuppressive activity of regulatory T cells (Tregs), leading to tumor growth and development (56). Liu et al. revealed that IL-17A increases PD-L1 expression through the p65/NRF1/miR-15b-5p axis, thereby promoting resistance to anti-PD-1 therapy. Blocking IL-17A improved the efficacy of anti-PD-1 treatment in murine models of MSS CRC (57). Another clinical analysis suggested that the activation of IL-17A signaling is associated with the failure of anti-PD-1 therapy in patients with colorectal cancer (58).

Prior research has shown that tumor cells release cytokines, vascular endothelial growth factors, and chemokines, which attract neutrophils into tumors. These neutrophils facilitate vascular invasion and contribute to the metastatic potential of tumor cells (59). Neutrophils also participate in creating an immunosuppressive microenvironment by releasing myeloperoxidase and arginase-1, and upregulating PD-L1. This, in turn, reduces the number of tumor-infiltrating lymphocytes (TIL) and leads to decreased effectiveness of immunotherapy (60). The correlation between peripheral blood NLR and clinical outcomes may be explained by the association between tumor-infiltrating lymphocytes and neutrophils, which results in reduced anti-tumor T-cell responses (61, 62).

As depicted in **Figure 4**, we observed changes in cytokine levels after treatment in both Cohorts. Cancer cells are the primary sources of cytokines, so successful treatment can lead to reductions in specific cytokines, as observed for IL-2, TNF-α, IFN-γ, and IL-17A in Cohort 2. However, patients treated with chemotherapy alone exhibited stabilization or an increase in levels of IL-4, IL-6, and IL-10 cytokines, which may suggest that the crucial cell compartments contributing to the presence of these cytokines might not be affected by chemotherapy, such as M2 macrophages in the tumor microenvironment (63, 64). Furthermore, the addition of ICIs increased concentrations of cytokines after treatment globally and appeared to counteract the effect of chemotherapy, which typically decreases cytokine levels. It is believed that cytokine levels reflect the immunosuppressive state to some extent, where a high level of cytokines indicates that the body is more sensitive to PD-1 antibodies (65). This finding is consistent with our observation that GC patients in Cohort 1 with more than 20% variation in IL-2 from baseline to the point of maximum remission had better OS.

## Conclusion

In conclusion, ongoing studies are actively investigating the predictive role of peripheral blood indicators in the effectiveness and prognosis of immunotherapy. However, comprehensive data on the use of Immune Checkpoint Inhibitors (ICIs) in advanced gastric cancer patients, both domestically and internationally, are still limited. Therefore, further prospective validation is required. To sum up, serum cytokines have varying significance in assessing the response of gastric cancer (GC) patients to anti-PD-1 therapy. Baseline levels of IL-2, IL-6, IL-17A, and Neutrophil-to-Lymphocyte Ratio (NLR), as well as changes in IL-2 levels over time, may serve as convenient predictive biomarkers for identifying GC patients who are likely to benefit from the addition of anti-PD-1 monoclonal antibodies to chemotherapy.

## Data availability statement

The raw data supporting the conclusions of this article will be made available by the authors, without undue reservation.

## Ethics statement

The studies involving humans were approved by Research and Clinical Trial Ethics Committee of the First Affiliated Hospital of Zhengzhou University. The studies were conducted in accordance with the local legislation and institutional requirements. The ethics committee/institutional review board waived the requirement of written informed consent for participation from the participants or the participants’ legal guardians/next of kin because The study was retrospective, with anonymous patient information obtained through the hospital record system, and all patients were deceased.

## Author contributions

YH: Data curation, Formal Analysis, Writing – original draft. XL: Validation, Visualization, Writing – original draft. YY: Investigation, Software, Writing – review & editing. HS: Conceptualization, Writing – review & editing. SW: Investigation, Writing – review & editing. MG: Funding acquisition, Methodology, Writing – review & editing.
