# Supplementary material for: Serum cytokines and neutrophil-to-lymphocyte ratio as predictive biomarkers of benefit from PD-1 inhibitors in gastric cancer
Source: Front Immunol. 2023 Oct 31;14:1274431. doi: 10.3389/fimmu.2023.1274431 (PMC10643875; doi:10.3389/fimmu.2023.1274431)
Supplement: Supplementary file 1 [file DataSheet_1.pdf]

Supplementary Material

Supplementary Tables:

Table S1: The median of baseline blood parameters.

median	IL-2	IL-4	IL-6	IL-10	TNF-α	IFN-γ	IL-17A	TLC	NLR	PLR	LMR
**Cohort 1**	1.96	2.37	12.06	2.86	1.87	2.11	8.20	1.31	2.60	149.23	3.46
**Cohort 2**	1.49	1.93	4.85	2.65	2.23	1.94	5.72	1.37	2.34	149.69	3.71

Table S2: The median of blood parameters after 2 cycles of treatment.

median	IL-2	IL-4	IL-6	IL-10	TNF-α	IFN-γ	IL-17A	TLC	NLR	PLR	LMR
**Cohort 1**	2.65	3.71	7.31	2.88	2.82	2.01	6.21	1.35	3.01	114.57	3.14
**Cohort 2**	2.32	2.49	7.27	3.03	2.45	2.61	4.61	1.36	1.89	134.81	2.66

Table S3: The cut-off value of baseline blood parameters calculated by the web-based software X-tile.

	cut-off value	IL-2	IL-4	IL-6	IL-10	TNF-α	IFN-γ	IL-17A	TLC	NLR	PLR	LMR
Cohort 1	OS	1.83	2.79	14.34	2.18	1.27	2.11	7.43	0.90	1.41	189.23	2.00
	PFS	1.42	2.91	17.39	2.18	1.15	1.50	5.68	1.20	2.31	111.83	3.12
Cohort 2	OS	1.51	0.91	3.60	4.54	3.40	1.41	5.30	2.03	2.92	282.76	2.91
	PFS	2.03	1.91	9.58	2.42	2.20	2.62	5.30	0.93	2.92	189.10	3.45

Table S4: The cut-off value of blood parameters after 2 cycles of treatment calculated by the web-based software X-tile.

	cut-off value	IL-2	IL-4	IL-6	IL-10	TNF-α	IFN-γ	IL-17A	TLC	NLR	PLR	LMR
Cohort 1	OS	2.71	2.21	15.35	3.21	2.71	3.78	15.31	1.63	2.81	114.57	4.63
	PFS	3.20	3.71	20.23	3.32	2.71	1.10	3.33	1.60	2.81	207.41	1.42
Cohort 2	OS	2.54	1.62	12.66	2.12	2.53	2.22	6.40	1.42	1.30	88.29	2.39
	PFS	1.40	3.81	8.38	3.72	1.28	2.22	6.40	1.82	1.89	153.00	2.97

Table S5: Characteristics of healthy controls and the GC groups.

Clinical characteristics		healthy control n=33	GC patients n=77	P value
**gender**	male	20(60.6)	53(68.8)	0.403
	female	13(39.4)	24(31.2)	
**age**	＜60	16(48.5)	40(51.9)	0.739
	≥60	17(51.5)	37(48.1)	

Table S6: Comparison of baseline blood parameters between GC patients and healthy individuals.

	Healthy individual		GC patients		P value
	median	Q1-Q3	median	Q1-Q3	
**IL-2（pg/mL）**	1.82	1.28-2.40	1.74	1.20-2.60	0.924
**IL-4（pg/mL）**	2.17	1.80-2.72	1.99	0.88-3.31	0.655
**IL-6（pg/mL）**	**3.86**	2.15-4.48	**6.10**	3.96-14.27	**<0.0001**
**IL-10（pg/mL）**	**1.86**	1.13-2.46	**2.69**	1.84-3.82	**<0.0001**
**TNF-α（pg/mL）**	**1.41**	0.41-2.54	**1.94**	1.30-2.74	**0.021**
**IFN-γ（pg/mL）**	1.90	1.46-2.72	2.06	1.46-2.61	0.774
**IL-17A（pg/mL）**	4.71	1.02-6.82	8.20	2.78-9.44	0.075
**TLC（10^9）**	**1.97**	1.61-2.24	**1.32**	1.02-1.74	**<0.0001**
**NLR**	**1.75**	1.31-2.46	**2.34**	1.53-3.61	**0.0085**
**PLR**	**121.10**	99.28-136.9	**149.20**	109.7-216.3	**0.0034**
**LMR**	**5.00**	3.95-6.27	**3.48**	2.34-4.25	**<0.0001**

Table S7: Correlation between baseline blood indexes and clinical features in GC patients, median (Q1, Q3).

characteristics		IL-2	IL-4	IL-6	IL-10	TNF-α	IFN-γ	IL-17A	TLC	NLR	PLR	LMR
gender	male	1.64 (1.11-2.48)	1.91(0.83-3.11)	**5.81(3.51-12.36)**	2.69(1.88-3.78)	2(1.39-2.85)	2.04(1.42-2.65)	5.31(2.46-8.98)	1.31(1.02-1.7)	**2.5(1.76-4.48)**	**157.27(110.88-260.72)**	3.38(2.1-4.12)
	female	1.79(1.48-3.05)	2.78(1.25-4.08)	**12.09(4.95-22.45)**	2.59(1.78-5.22)	1.6(0.99-2.48)	2.07(1.69-2.58)	6.02(3.82-9.87)	1.58(0.95-1.81)	**1.45(1.07-2.62)**	**115.51(102.41-170)**	3.85(3.17-4.77)
**P**				**0.032**						**0.003**	**0.046**	
smoke	no	1.79(1.18-2.58)	2.3(0.99-3.22)	6.19(3.54-14.57)	2.79(1.97-3.85)	1.95(1.26-2.91)	2.07(1.68-2.57)	5.66(3.77-9.66)	1.31(1-1.66)	2.56(1.55-4.12)	149.58(111.35-248.01)	3.36(2.22-4.15)
	yes	1.71(1.21-2.6)	1.75(0.8-3.33)	6.08(4.58-14.19)	2.49(1.8-3.78)	1.94(1.3-2.63)	1.74(1.18-2.77)	6.86(2.01-9.04)	1.4(1.04-2.08)	2.07(1.44-3.35)	140.91(103.76-189.1)	3.87(2.39-4.46)
**P**												
family	no	1.83(1.11-2.76)	1.93(0.82-3.21)	**8.7(4.58-15.66)**	2.82(1.81-3.87)	2.04(1.3-2.91)	2.07(1.41-2.62)	5.68(2.34-9.62)	1.31(0.93-1.7)	2.39(1.6-4.01)	151.33(109.92-226.92)	3.46(2.3-4.05)
	yes	1.51(1.37-2.08)	2.17(0.99-4.3)	**4.06(2.98-6.68)**	2.47(1.95-3.47)	1.6(0.99-2.56)	1.93(1.69-2.4)	5.68(3.06-10.36)	1.49(1.21-1.87)	1.9(1.44-3.03)	124.51(100.79-167.36)	4.17(2.58-5.55)
**P**				**0.012**								
disease	no	1.74(1.29-2.48)	1.93(0.88-3.06)	6.1(3.98-15.46)	2.65(1.91-3.82)	1.97(1.45-2.66)	2.06(1.52-2.55)	5.31(3.05-7.89)	1.4(1.1-1.79)	2.31(1.59-3.2)	148.53(110.88-193.36)	**3.78(2.72-4.4)**
	yes	1.79(1.02-2.84)	2.7(0.87-4.81)	6.25(3.51-12.49)	2.96(1.73-4.25)	1.78(1.03-3.07)	2(1.37-3.5)	9.09(2.09-18.68)	1.28(0.8-1.7)	2.78(1.37-5.59)	176.73(100.76-321.6)	**2.62(2.09-3.84)**
**P**												**0.028**
stage	I-II	1.42(1.08-3.2)	1.73(0.8-2.61)	5.53(2.57-16.97)	2.8(2.05-3.79)	2.09(1.09-2.97)	1.93(1.49-2.43)	4.51(2.17-7.97)	1.35(0.87-1.65)	2.89(1.89-4.12)	130.27(102.43-257.74)	3.25(1.97-4.27)
	III-IV	1.86(1.36-2.59)	2.32(0.91-3.31)	6.42(4.42-14.19)	2.65(1.8-3.87)	1.92(1.42-2.69)	2.08(1.43-2.62)	5.82(3.19-9.79)	1.31(1.04-1.79)	2.15(1.44-3.44)	157.27(112.92-209.38)	3.71(2.37-4.26)
**P**												
ecog	≤2	1.71(1.18-2.67)	1.93(0.91-3.21)	6.42(3.99-14.34)	2.69(1.92-3.79)	1.97(1.3-2.69)	2.07(1.5-2.59)	5.64(2.65-8.87)	**1.4(1.1-1.82)**	2.31(1.46-3.35)	140.72(103.05-205.83)	3.48(2.3-4.24)
	＞2	1.79(1.24-2.25)	2.46(0.79-3.9)	6.05(3.51-13.63)	2.47(1.66-4.07)	1.56(1.26-2.83)	1.98(1.36-2.94)	8.22(3.17-14.57)	**1.1(0.81-1.38)**	2.76(2.12-4.5)	176.73(135.2-291.42)	3.64(2.32-5.38)
**P**								**0.001**	**0.048**			
surgery	no	50(25-75)	1.49(1.1-2.36)	1.75(0.8-3.34)	6.1(3.97-13.7)	2.58(1.87-3.54)	**1.89(1.1-2.55)**	1.84(1.41-2.25)	36.4(34-40)	**1.4(1.2-1.7)**	2.07(1.41-2.86)	149.47(111.83-209.38)
	yes	2.16(1.51-2.8)	2.59(1.31-3.31)	7.39(3.86-14.67)	2.87(1.79-4.13)	2.28(1.59-2.96)	**2.41(1.88-3.06)**	6.99(3.69-10.63)	1.21(0.9-1.87)	**2.72(2.04-5.01)**	148.88(108.07-257.66)	3.34(2.04-3.88)
**P**							**0.009**	**0.037**		**0.017**		
age	＞60	1.71(1.19-2.26)	1.93(0.81-3)	**8.72(4.59-16.99)**	2.86(2.04-3.87)	1.72(1.25-2.74)	2.06(1.48-2.52)	5.3(2.25-9.15)	1.24(0.92-1.81)	2.55(1.69-4.48)	149.23(109.72-246.07)	3.38(2.15-3.88)
	≤60	1.92(1.22-2.75)	2.35(0.94-3.45)	**5.25(3.18-12.05)**	2.5(1.75-3.66)	2.15(1.4-2.86)	2.08(1.44-2.98)	6(2.97-10.27)	1.56(1.15-1.7)	2.17(1.47-3.07)	146.12(105.78-216.78)	3.89(2.41-5.08)
**P**				**0.048**								

Table S8: Univariate and Multivariate analysis for PFS and OS of Cohort 1 (blood parameters after 2 cycles of treatment) grouped by median.

Characteristics	OS				PFS			
	univariate analysis		multivariate analysis		univariate analysis		multivariate analysis	
	HR(95% CI)	P	HR(95% CI)	P	HR(95% CI)	P	HR(95% CI)	P
IL-2	**0.404(0.198-0.826)**	**0.013**	**0.464(0.221-0.971)**	**0.042**	0.639(0.333-1.225)	0.177	-	-
IL-4	0.629(0.335-1.182)	0.150	-	-	0.769(0.412-1.435)	0.409	-	-
IL-6	**2.677(1.349-5.309)**	**0.005**	**2.136(1.004-4.544)**	**0.049**	**1.907(0.993-3.662)**	**0.053**	**4.426(1.857-10.549)**	**0.001**
IL-10	0.854(0.452-1.613)	0.627	-	-	0.661(0.337-1.295)	0.227	-	-
TNF-α	0.737(0.392-1.384)	0.342	-	-	0.644(0.339-1.224)	0.179	-	-
IFN-γ	1.145(0.614-2.135)	0.671	-	-	1.061(0.568-1.981)	0.853	-	-
IL-17A	1.663(0.88-3.146)	0.118	-	-	**2.171(1.144-4.121)**	**0.018**	**4.745(2.008-11.217)**	**0.000**
TLC	0.856(0.458-1.599)	0.625	-	-	0.828(0.444-1.545)	0.554	-	-
NLR	**2.25(1.165-4.344)**	**0.016**	1.656(0.791-3.467)	0.181	**2.207(1.166-4.177)**	**0.015**	1.423(0.743-2.726)	0.288
PLR	1.065(0.571-1.987)	0.843	-	-	1.553(0.826-2.922)	0.172	-	-
LMR	0.884(0.473-1.651)	0.698	-	-	0.696(0.371-1.307)	0.260	-	-
gender	0.921(0.479-1.768)	0.804	-	-	0.875(0.459-1.667)	0.685	-	-
age（year）	0.74(0.39-1.403)	0.357	-	-	0.968(0.504-1.86)	0.922	-	-
ECOG（＞2）	**10.172(3.453-29.966)**	**0.000**	**10.019(3.072-32.68)**	**0.000**	1.659(0.723-3.81)	0.233	-	-
TNM stage（III-IV）	1.267(0.526-3.055)	0.598	-	-	0.953(0.391-2.322)	0.915	-	-
surgery history	1.33(0.699-2.529)	0.385	-	-	0.957(0.512-1.79)	0.891	-	-
other chronic disease	1.001(0.484-2.069)	0.999	-	-	1.072(0.521-2.208)	0.850	-	-
smoked	0.903(0.47-1.734)	0.758	-	-	0.97(0.508-1.852)	0.927	-	-
family cancer history	1.373(0.563-3.349)	0.486	-	-	0.765(0.319-1.833)	0.548	-	-

Cohort 1 blood parameters after 2 cycles of treatment were grouped by the median.

Table S9: Univariate and Multivariate analysis for PFS and OS of Cohort 1 (blood parameters after 2 cycles of treatment) grouped by cut-off value.

Characteristics	OS				PFS			
	univariate analysis		multivariate analysis		univariate analysis		multivariate analysis	
	HR(95% CI)	P	HR(95% CI)	P	HR(95% CI)	P	HR(95% CI)	P
IL-2	**0.334(0.158-0.703)**	**0.004**	**0.3(0.136-0.663)**	**0.003**	**0.394(0.183-0.852)**	**0.018**	**0.243(0.102-0.577)**	**0.001**
IL-4	0.433(0.147-1.279)	0.130	-	-	0.769(0.412-1.435)	0.409	-	-
IL-6	**4.062(1.804-9.145)**	**0.001**	**9.01(2.733-29.702)**	**0.000**	**6.54(2.317-18.464)**	**0.000**	**5.731(1.795-18.295)**	**0.003**
IL-10	0.805(0.413-1.568)	0.524	-	-	1.19(0.618-2.29)	0.603	-	-
TNF-α	0.699(0.373-1.312)	0.266	-	-	0.6(0.315-1.139)	0.118	-	-
IFN-γ	1.906(0.822-4.421)	0.133	-	-	2.085(0.788-5.515)	0.139	-	-
IL-17A	**3.617(1.419-9.223)**	**0.007**	**5.756(2.017-16.42)**	**0.001**	0.635(0.309-1.308)	0.218	-	-
TLC	2.177(1.126-4.209)	0.021	2.667(0.837-8.502)	0.097	**2.305(1.185-4.483)**	**0.014**	1.214(0.471-3.131)	0.689
NLR	**0.424(0.22-0.816)**	**0.010**	1.796(0.498-6.482)	0.371	**0.373(0.19-0.733)**	**0.004**	0.663(0.248-1.769)	0.411
PLR	0.683(0.366-1.275)	0.231	-	-	0.557(0.232-1.339)	0.191	-	-
LMR	1.493(0.752-2.965)	0.252	-	-	**2.801(0.964-8.143)**	**0.059**	1.371(0.44-4.267)	0.586
gender	0.921(0.479-1.768)	0.804	-	-	0.875(0.459-1.667)	0.685	-	-
age（year）	0.74(0.39-1.403)	0.357	-	-	0.968(0.504-1.86)	0.922	-	-
ECOG（＞2）	**10.172(3.453-29.966)**	**0.000**	23.868(5.091-111.894)	0.000	1.659(0.723-3.81)	0.233	-	-
TNM stage（III-IV）	1.267(0.526-3.055)	0.598	-	-	0.953(0.391-2.322)	0.915	-	-
surgery history	1.33(0.699-2.529)	0.385	-	-	0.957(0.512-1.79)	0.891	-	-
other chronic disease	1.001(0.484-2.069)	0.999	-	-	1.072(0.521-2.208)	0.850	-	-
smoked	0.903(0.47-1.734)	0.758	-	-	0.97(0.508-1.852)	0.927	-	-
family cancer history	1.373(0.563-3.349)	0.486	-	-	0.765(0.319-1.833)	0.548	-	-

Cohort 1 blood parameters after 2 cycles of treatment were grouped by cut-off value.

Table S10: Univariate and multivariate analysis for PFS and OS of Cohort 2 (blood parameters after 2 cycles of treatment) grouped by median.

Clinical characteristics	OS				PFS			
	univariate analysis		multivariate analysis		univariate analysis		multivariate analysis	
	HR (95% CI)	P	HR (95% CI)	P	HR (95% CI)	P	HR (95% CI)	P
IL-2	0.818(0.408-1.641)	0.572	-	-	0.969(0.497-1.892)	0.927	-	-
IL-4	0.741(0.376-1.46)	0.386	-	-	0.751(0.369-1.528)	0.429	-	-
IL-6	1.109(0.568-2.164)	0.761	-	-	0.817(0.418-1.598)	0.555	-	-
IL-10	0.715(0.364-1.404)	0.330	-	-	0.602(0.301-1.207)	0.153	-	-
TNF-α	0.981(0.499-1.927)	0.955	-	-	1.507(0.762-2.982)	0.239	-	-
IFN-γ	1.32(0.678-2.572)	0.415	-	-	0.893(0.454-1.755)	0.742	-	-
IL-17A	1.64(0.821-3.276)	0.161	-	-	1.617(0.82-3.188)	0.166	-	-
TLC	**1.874(0.946-3.71)**	**0.072**	1.487(0.711-3.111)	0.292	1.259(0.64-2.478)	0.505	-	-
NLR	1.485(0.722-3.055)	0.282	-	-	0.811(0.413-1.591)	0.542	-	-
PLR	0.605(0.306-1.196)	0.149	-	-	0.984(0.504-1.922)	0.963	-	-
LMR	1.406(0.719-2.75)	0.319	-	-	1.501(0.763-2.952)	0.239	-	-
gender	1.204(0.46-3.154)	0.705	-	-	1.209(0.463-3.161)	0.698	-	-
age	0.731(0.372-1.439)	0.365	-	-	1.074(0.549-2.101)	0.836	-	-
ECOG,（＞2）	1.638(0.701-3.831)	0.255	-	-	1.867(0.786-4.435)	0.157	-	-
TNM stage, （III-IV）	1.047(0.528-2.077)	0.895	-	-	0.86(0.436-1.694)	0.662	-	-
surgery history	**0.111(0.036-0.34)**	**0.000**	**0.148(0.042-0.517)**	**0.003**	0.493(0.209-1.161)	0.106	-	-
other chronic basic disease	1.155(0.54-2.47)	0.711	-	-	0.771(0.367-1.623)	0.494	-	-
smoked	**1.992(0.96-4.134)**	**0.064**	1.224(0.503-2.974)	0.656	1.614(0.787-3.307)	0.191	-	-
family cancer history	0.767(0.332-1.77)	0.534	-	-	0.761(0.323-1.791)	0.532	-	-

Cohort 2 blood parameters after 2 cycles of treatment were grouped by the median.

Table S11: Univariate and multivariate analysis for PFS and OS of Cohort 2 (blood parameters after 2 cycles of treatment) grouped by cut-off value.

Clinical characteristics	OS				PFS			
	univariate analysis		multivariate analysis		univariate analysis		multivariate analysis	
	HR (95% CI)	P	HR (95% CI)	P	HR (95% CI)	P	HR (95% CI)	P
IL-2	0.726(0.355-1.485)	0.380	-	-	1.461(0.726-2.938)	0.288	-	-
IL-4	0.578(0.268-1.245)	0.161	-	-	0.81(0.4-1.638)	0.557	-	-
IL-6	0.7(0.345-1.421)	0.324	-	-	1.72(0.738-4.009)	0.209	-	-
IL-10	1.317(0.537-3.229)	0.548	-	-	0.603(0.302-1.206)	0.153	-	-
TNF-α	1.5(0.614-3.668)	0.374	-	-	1.378(0.695-2.732)	0.358	-	-
IFN-γ	0.73(0.298-1.789)	0.492	-	-	1.513(0.715-3.202)	0.279	-	-
IL-17A	1.342(0.685-2.628)	0.391	-	-	**1.856(0.928-3.709)**	**0.080**	-	-
TLC	**7.71(2.389-24.883)**	**0.001**	2.626(0.595-11.586)	0.202	0.596(0.241-1.474)	0.262	-	-
NLR	1.458(0.641-3.315)	0.368	-	-	1.805(0.791-4.122)	0.161	-	-
PLR	1.003(0.409-2.462)	0.994	-	-	1.661(0.581-4.746)	0.344	-	-
LMR	0.904(0.443-1.843)	0.781	-	-	1.745(0.878-3.47)	0.112	-	-
gender	1.204(0.46-3.154)	0.705	-	-	1.209(0.463-3.161)	0.698	-	-
age	0.731(0.372-1.439)	0.365	-	-	1.074(0.549-2.101)	0.836	-	-
ECOG,（＞2）	1.638(0.701-3.831)	0.255	-	-	1.867(0.786-4.435)	0.157	-	-
TNM stage,（III-IV）	1.047(0.528-2.077)	0.895	-	-	0.86(0.436-1.694)	0.662	-	-
surgery history	**0.111(0.036-0.34)**	**0.000**	**0.206(0.047-0.903)**	**0.036**	0.493(0.209-1.161)	0.106	-	-
other chronic basic disease	1.155(0.54-2.47)	0.711	-	-	0.771(0.367-1.623)	0.494	-	-
smoked	**1.992(0.96-4.134)**	**0.064**	1.146(0.456-2.876)	0.772	1.614(0.787-3.307)	0.191	-	-
family cancer history	0.767(0.332-1.77)	0.534	-	-	0.761(0.323-1.791)	0.532	-	-

Cohort 2 blood parameters after 2 cycles of treatment were grouped by cut-off value.
